# Enteral Nutrition Interruptions in the Intensive Care Unit: A Systematic Review of Frequency, Causes, and Nutritional Implications

**DOI:** 10.7759/cureus.81834

**Published:** 2025-04-07

**Authors:** Sjaak Pouwels, Monica M van Nieuwkoop, Dharmanand Ramnarain

**Affiliations:** 1 Intensive Care Medicine, Elisabeth-Tweesteden Hospital, Tilburg, NLD; 2 Surgery, Marien Hospital Herne, University Hospital of Ruhr University Bochum, Herne, DEU; 3 Critical Care Medicine, Elisabeth-Tweesteden Hospital, Tilburg, NLD

**Keywords:** critical care, enteral nutrition, enteral nutrition interruption, intensive care, malnutrition, underfeeding, undernutrition

## Abstract

Enteral nutrition interruptions (ENIs) are a major cause of inadequate nutrition goals in critically ill patients. The aim of this systematic review was to provide an update on the various clinical and logistical reasons for ENIs and observe their nutritional implications. PubMed, MEDLINE, Embase, and The Cochrane Library were searched from the inception of each database until March 11, 2024. For data extraction, a structured checklist was used. The initial literature search yielded 522 results. In total, 26 studies were included, comprising 3067 participants. Among the included studies, there were 20 prospective studies, two before-and-after studies, one RCT, and three retrospective studies. The main reasons for ENI were high gastric residual volumes, nasogastric tube dysfunction, and diagnostic and surgical procedures. In conclusion, although the nutritional management of critically ill patients in the ICU has improved drastically, ENIs remain a major problem in clinical practice. Future research should consider different treatments and ICU protocols. Additionally, there is a need for standardized ENI definitions and standardized reporting of the evaluation of energy and/or protein requirements, objectively determining adequate intake, and reporting the causes, frequency, and duration of ENIs.

## Introduction and background

Enteral nutrition (EN) is part of standard care in the intensive care unit (ICU) when oral feeding is impossible or when a patient does not achieve their nutritional goals within a reasonable time frame [1-3]. EN is associated with improvements in gastrointestinal mucosa integrity, immune function, and tissue repair responses. It has also been associated with a reduction in nosocomial infections, ICU length of stay, and healthcare costs [4].

However, a recent study showed that the desired intake of EN was successful in 37% of the feeding days [5]. Another study showed that only 29% of the patients received adequate energy intake and only 44% received adequate protein intake during the feeding days with EN [6]. The American Society for Parenteral and Enteral Nutrition (ASPEN) and the Society of Critical Care Medicine (SCCM) conducted a study that showed less than half of critically ill patients ever reach their target energy intake during their ICU stay [7]. A common reason for inadequacy in meeting nutrition goals is an enteral nutrition interruption (ENI). ENIs are common among critically ill ICUs; however, the exact incidence varies among studies [7]. ENIs can be broadly characterized by patient-related factors (e.g., gastrointestinal dysfunction and unstable hemodynamics) or procedure-related factors (e.g., planned surgery, transport to computed tomography (CT), or magnetic resonance imaging scan (MRI)) [7].

Despite extensive research on ENIs, their causes, frequency, and duration vary, partly due to the diversity in the hospital protocols in different countries. ENIs are frequently described in the literature since inadequacy of caloric and protein increases the risk of malnutrition [1]. Malnourished patients have a higher risk of complications and longer stay in the hospital, which might eventually lead to higher mortality and healthcare costs [8,9].

Kim et al. [6] have written a systematic review on the adequacy of EN back in 2012. They showed that there is no 'easy answer' to this complex matter. The aim of this systematic review is to give an overview of the frequency, causes, and nutritional implications of ENIs. Secondly, we will give an update on the evidence regarding EN adequacy in critically ill patients. Our research questions were the following: 1) What is the frequency of ENIs in the literature? 2) What are the causes/reasons for ENIs in ICU patients? and 3) What are the possible implications of ENIs in ICU patients?

## Review

Materials and methods

A multi-database systematic literature search was done. The patient population of interest was all critically ill adult patients in the ICU receiving EN, The intervention of interest was EN, which needed to be started within 48 hours after ICU admission, and outcome measures of interest were initiation time of EN, daily goals of calories and protein, daily calories and protein delivered, and duration and causes of ENIs. Studies were excluded when patients received partial oral food intake, full food intake, or solely parenteral nutrition.

A database search was performed using the following databases: PubMed, MEDLINE, Embase, and The Cochrane Library. PubMed and The Cochrane Library were selected because most of the relevant literature for our systematic review is indexed in these databases. Secondly, we chose to additionally search Embase and Medline to find possible conference abstracts important for our systematic review. The following search words were used and were modified for each database: [(Intensive Care OR Critical Care) AND (Enteral Nutrition interruption OR enteral nutrition intolerance)]. We were only interested in adult ICU patients, so we used the filters [Humans] and [Adult 18+ Years]. All the databases were searched from the earliest date of each database (i.e., the date of inception) until 11 March 2024.

Studies were separately screened and selected on the basis of title and abstract. After the primary search process, both authors reviewed the studies that were selected and defined whether they were eligible for inclusion. The process of this systematic review was done in accordance with the Preferred Reporting Items for Systematic Reviews (PRISMA) guidelines [10]. For further eligible studies, cross-references were screened. All three authors read the full-text articles of the initial search strategy and filtered out any potentially relevant studies for this systematic review that were not included already. All disagreements were discussed with each other or with the senior author until a consensus was reached.

Randomized controlled trials (RCTs) and prospective and retrospective studies published in English, German, or Dutch were included in this systematic review. For data extraction, a structured checklist was used, including the frequency and duration of ENIs, nutritional adequacy, initiation time of EN (if possible), daily goals of calories and protein, and daily calories and protein delivered.

The methodological quality of the included studies was rated using the Newcastle-Ottawa scale (NOS) [1,10]. Stars awarded for each quality item serve as a quick visual assessment. Stars are awarded such that the highest quality studies are up to nine stars. The NOS assigns up to a maximum of nine points for the least risk of bias in three domains: (1) selection of study groups (4 points), (2) comparability of groups (2 points), and (3) ascertainment of exposure and outcomes (3 points) for case-control and cohort studies, respectively. Two authors (SP and MvN) separately assessed the NOS scale of the included studies. A Cohen’s kappa score was calculated to determine the level of agreement between authors MvN and SP. A Cohen’s kappa score <0.20 indicates a poor agreement, 0.21-0.40 a fair agreement, 0.41-0.60 a moderate agreement, 0.61-0.80 a good agreement, and 0.81-1.00 a very good agreement.

Statistical analysis

Due to inconsistent reporting of outcome measures and a small number of included studies (e.g., patients), a meta-analysis was not performed. Continuous variables were shown as mean ± standard deviation (SD) and categorical variables as frequency with percentages. Statistical Package for Social Sciences (SPSS, Chicago, IL, USA Version 20.0) was used to prepare a database and for statistical analysis.

Results

**Figure 1:**
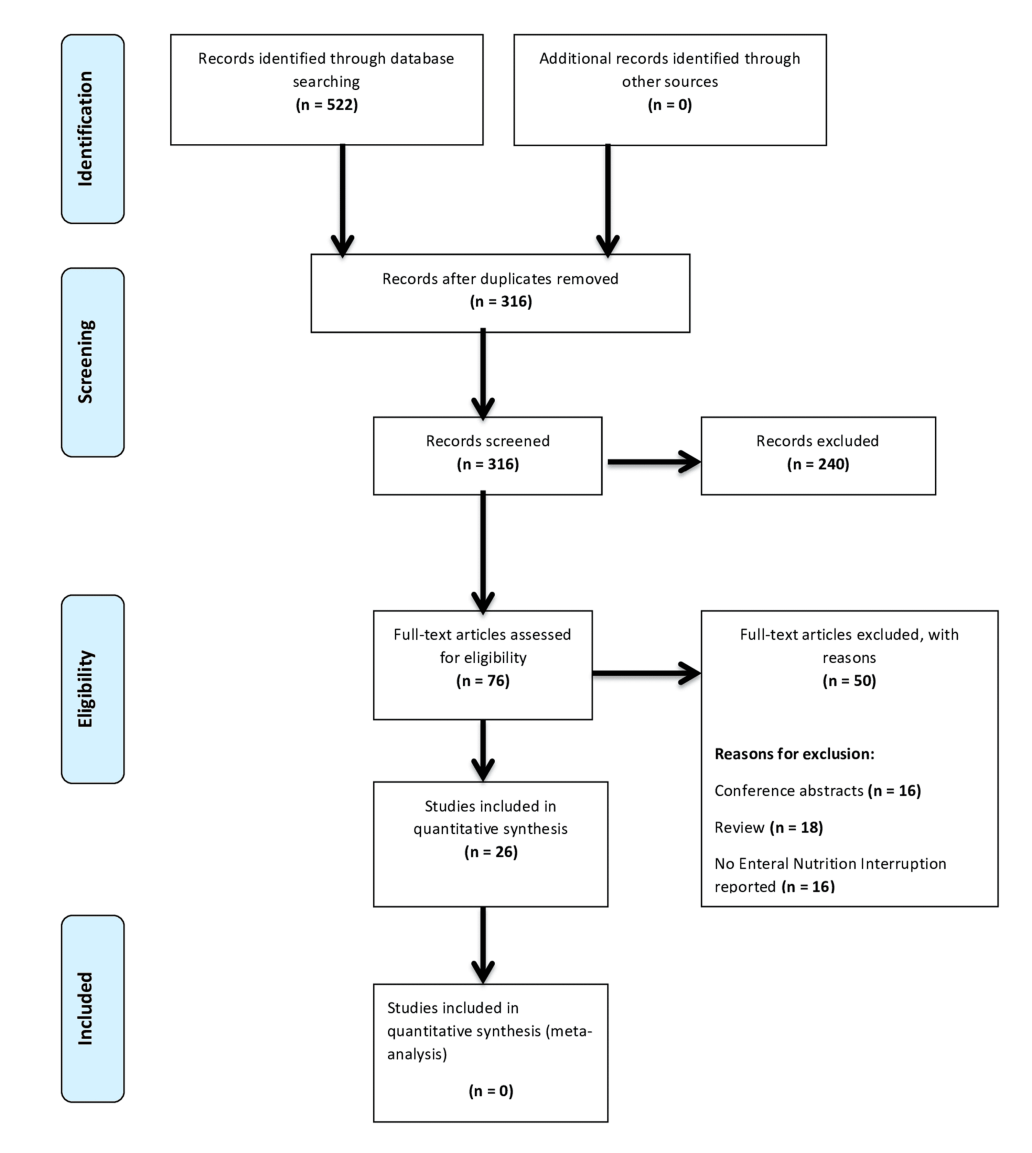
PRISMA flowchart of the included studies PRISMA: Preferred Reporting Items for Systematic Reviews

The initial literature search produced 522 results, including 240 duplicates. After screening for title and abstract, 76 potentially relevant studies were found and underwent a full-text critical appraisal. Of these studies, a total of 50 were excluded due to the following reasons: 16 abstracts were not available, 18 were reviews, and in the remaining studies, there were no ENIs or related events reported. In total, 26 studies [3,5,6,11-33] were included in our systematic review. Figure 1 shows the PRISMA flowchart of the search strategy.

The methodological quality of the included studies ranged from moderate to good, as indicated by the NOS (Table 1) [17]. A Cohen’s kappa of 0.75 reflected a good agreement between authors SP and MvN. 

**Table 1:** Assessment of methodological quality using the NOS ^*^The study suffices for this criterion point. ^**^The study suffices for these two criterion points. For criteria points S1-S4 and O1-O3, it is possible to achieve 1 star. For criterion C1, it is possible to achieve 2 stars. S1: representativeness; S2: selection; S3: ascertainment; S4: demonstration; C1: comparability; O1: outcome selection; O2: outcome follow-up; O3: adequacy; NOS: Newcastle-Ottawa scale

Criteria	S1	S2	S3	S4	C1	O1	O2	O3	T
Salciute-Simene et al. [1]	*	-	*	-	*	*	*	*	6
Wesselink et al. [11]	*	*	*	-	**	*	*	*	8
Williams et al. [12]	*	*	*	-	**	*	*	*	8
Segaran et al. [13]	*	*	*	*	**	*	*	*	9
Yip et al. [14]	*	*	*	*	**	*	*	*	9
Stechmiller et al. [15]	*	-	*	-	**	*	-	*	6
Ritter et al. [16]	*	-	*	-	*	*	*	*	6
Pinilla et al. [17]	*	*	*	-	*	*	*	*	7
Peev et al. [18]	*	*	*	-	*	*	*	*	7
Passier et al. [19]	*	-	*	-	**	*	*	*	7
Lee et al. [20]	*	-	*	-	**	*	*	*	7
Kozeniecki et al. [21]	*	-	*	-	**	*	*	*	7
Kim et al. [6]	*	*	*	-	**	*	*	*	8
Engel et al. [22]	*	-	*	-	**	*	*	*	7
De Jonghe et al. [23]	*	-	*	-	*	*	*	*	6
Chapple et al. [5]	*	*	*	-	**	*	*	*	8
Binnekade et al. [24]	*	*	*	-	**	*	*	*	8
Adam et al. [25]	*	*	*	*	**	*	*	*	9
Petros et al. [26]	*	*	*	*	**	*	*	*	9
Kim et al. [27]	*	-	*	-	**	*	-	*	6
Reid et al. [28]	*	-	*	-	*	*	*	*	6
McClave et al. [29]	*	*	*	-	*	*	*	*	7
Elpern et al. [30]	*	*	*	-	*	*	*	*	7
O'Leary-Kelley et al. [31]	*	-	*	-	**	*	*	*	7
Rice et al. [32]	*	-	*	-	**	*	*	*	7
O'Meara et al. [33]	*	-	*	-	**	*	*	*	7

Baseline characteristics

There were 20 prospective studies among the included studies, two before-and-after studies, one RCT, and three retrospective studies (Table 2). A total of 3067 participants were included in this systematic review. Only 20 studies reported the number of males and females: 1626 males (of 2617 participants, 62%) and 991 females (38%). Finally, only seven studies reported baseline anthropometric variables [3,5,16,27,29,31,32].

**Table 2:** Baseline characteristics of the included studies B&A: before-and-after; POCT: prospective observational cohort study; ROCT: retrospective observational cohort study; RCT: randomized controlled trial; SD: standard deviation

Study	Year	Type of study	N (total)	N (intervention)	N (control)	Males (N(%))	Females (N/%))	Length (mean±SD)	Weight (mean±SD)	BMI (mean±SD)
Stechmiller [11]	1994	POCT	52	52		24 (46.2%)	28 (53.8%)			
Adam [12]	1997	POCT	193	193						
McClave [13]	1999	POCT	44							
Pinilla [15]	2001	RCT	80	36	44	44 (55%)	36 (45%)			
De Jonghe [14]	2001	POCT	51	51		28 (54.9%)	23 (45.1%)			
Engel [16]	2003	POCT	60	39	21	43 (71.7%)	17 (28.3%)	177±9	83±14	
Elpern [17]	2004	POCT	39							
Binnekade [18]	2005	ROCT	404	404		256 (63.4%)	148 (36.6%)			
O'Leary-Kelley [19]	2005	POCT	60							
Rice [20]	2005	POCT	55							
Petros [21]	2006	POCT	61	61		35 (57.4%)	26 (42.6%)			
Reid [22]	2006	POCT	32	32		15 (46.9%)	17 (53.1%)			
O'Meara [23]	2008	POCT	59							
Kim [24]	2010	POCT	47	47		26 (55.3%)	21 (44.7%)			
Kim [6]	2012	POCT	34	13	21	17 (50%)	17 (50%)			
Williams [26]	2013	B&A study	653	338	315	424 (65%)	229 (35%)			
Yip [27]	2013	POCT	77	77		49 (63.6%)	28 (36.4%)		68.2 (±11.8)	
Passier [25]	2013	ROCT	41	41		24 (59%)	17 (41%)			
Peev [28]	2015	POCT	94	64	30	67 (71%)	27 (29%)			
Segaran [30]	2016	B&A study	22	11	11	14 (64%)	8 (36%)			
Kozeniecki [29]	2016	ROCT	78	78		32 (41%)	46 (59%)			28 (23-32)
Chapple [5]	2016	POCT	37	37		32 (86.5%)	5 (13.5%)			26.7 (19.8-39.5)
Wesselink [32]	2018	ROCT	443	241	202	280 (63.2%)	163 (36.8%)		80 (70-92)	26.4 (24.0-30.8)
Lee [31]	2018	POCT	148	81	67	80 (54.1%)	68 (45.9%)	1.64±0.08	71.48±18.28	26.74±6.65
Ritter [33]	2019	POCT	130	130		83 (63.8%)	47 (36.2%)			
Salciute-Simene [3]	2021	POCT	73	73		53 (72.6%)	20 (27.4%)			28.5 (±8)

Enteral nutrition interruptions

Table 3 shows the number of patients admitted for medical and surgical disciplines, the total occurrence of ENIs, and the mean duration. Only 10 studies determined whether patients were admitted to the ICU for either a medical or a surgical specialty. The total number of ENI episodes was reported in 18 of the 26 included studies, which represents 4495 episodes of ENI. Only six studies reported the mean duration of the ENI, which ranges between 6.5 and 22 hours [3,5,11,25,26,31].

**Table 3:** Number of patients admitted for medical and surgical disciplines, the total occurrence of ENIs, and the mean duration ENI: enteral nutrition interruption; NR: not reported; H: hours; IG: intervention group; CG: control group; B&A: before-and-after; POCT: prospective observational cohort study; ROCT: retrospective observational cohort study; RCT: randomized controlled trial; SD: standard deviation

Study	Year	Type of study	N (%) (medical)	N (%) (surgical)	ENI (N)	Duration (h) IG (range or mean±SD)	Duration (h) CG (range or mean±SD)
Stechmiller [11]	1994	POCT	38 (73.1%)	14 (26.9%)	183	19.65 (4-96)	
Adam [12]	1997	POCT			479		
McClave [13]	1999	POCT					
Pinilla [15]	2001	RCT	41 (51.2%)	39 (48.8%)	55		
De Jonghe [14]	2001	POCT			94		
Engel [16]	2003	POCT			236		
Elpern [17]	2004	POCT					
Binnekade [18]	2005	ROCT	112 (27.7%)	292 (72.3%)			
O'Leary-Kelley [19]	2005	POCT					
Rice [20]	2005	POCT			79		
Petros [21]	2006	POCT			241		
Reid [22]	2006	POCT					
O'Meara [23]	2008	POCT	NR	NR	NR	NR	NR
Kim [24]	2010	POCT			124		
Kim [6]	2012	POCT					
Williams [26]	2013	B&A study	516 (79%)	137 (21%)	1569	22 (12-42)	22 (12-42)
Yip [27]	2013	POCT			72		
Passier [25]	2013	ROCT	10 (24.4%)	31 (75.6%)	121	6.5 (±6.2)	
Peev [28]	2015	POCT			106		
Segaran [30]	2016	B&A study			126		
Kozeniecki [29]	2016	ROCT			198		
Chapple [5]	2016	POCT	0 (0%)	37 (100%)	309	8.8 (3.4)	
Wesselink [32]	2018	ROCT	340 (76.7%)	103 (23.3%)			
Lee [31]	2018	POCT	116 (78.4%)	32 (21.6%)	332	12.6	
Ritter [33]	2019	POCT	66 (50.8)	64 (49.2%)	40		
Salciute-Simene [3]	2021	POCT	43 (59%)	30 (41%)	131	12 (6-24)	

Table 4 and Table 5 give an overview of the reported diagnostic procedures, intubations and extubations, and surgical procedures causing ENIs. In several studies, as shown in Table 4 and Table 5, it is not exactly specified that diagnostic or radiological investigations caused an ENI. The same occurs in several studies that report airway procedures as a causing factor for ENI but do not specify what exactly occurred.

**Table 4:** Diagnostic procedures causing ENIs ^*^Not specified Values are displayed as percentage or as frequency and percentage (N (%)) ENI: enteral nutrition interruption; CT: computed tomography; MRI: magnetic resonance imaging; OGD: esophagogastroduodenoscopy; B&A: before-and-after; POCT: prospective observational cohort study; ROCT: retrospective observational cohort study; RCT: randomized controlled trial

Study	Year	Type of study	CT	MRI	OGD	Colonoscopy	Bronchoscopy
Stechmiller [11]	1994	POCT	16 (9%)				
Adam [12]	1997	POCT					
McClave [13]	1999	POCT	27% due to radiology*	27% due to radiology*			
Pinilla [15]	2001	RCT	4 (7.3%)				
De Jonghe [14]	2001	POCT	Diagnostic procedures 25 (26.6%)*				
Engel [16]	2003	POCT					
Elpern [17]	2004	POCT					
Binnekade [18]	2005	ROCT					
O'Leary-Kelley [19]	2005	POCT	13.3% due to radiology*	13.3% due to radiology*			
Rice [20]	2005	POCT					
Petros [21]	2006	POCT	85 (35.4%) diagnostic procedures + 63 (26%) therapeutic procedures*				
Reid [22]	2006	POCT	3% due to radiology*	3% due to radiology*			
O'Meara [23]	2008	POCT	4.5% due to radiology*	4.5% due to radiology*			
Kim [24]	2010	POCT					
Kim [6]	2012	POCT					
Williams [26]	2013	B&A study	145 (9.2%)	95 (6.0%)			21 (1.3%)
Yip [27]	2013	POCT					
Passier [25]	2013	ROCT					
Peev [28]	2015	POCT					
Segaran [30]	2016	B&A study					
Kozeniecki [29]	2016	ROCT	12 (6.1%)				
Chapple [5]	2016	POCT					
Wesselink [32]	2018	ROCT					
Lee [31]	2018	POCT	38 (11.4%)				
Ritter [33]	2019	POCT					
Salciute-Simene [3]	2021	POCT	8 (6%)	1 (0.8%)	1 (0.8%)	1 (0.8%)	1 (0.8%)

**Table 5:** Intubation/extubation and surgical procedures causing ENIs Values are displayed as percentage or as frequency and percentage (N (%)) ENI: enteral nutrition interruption; NR: not reported; B&A: before-and-after; POCT: prospective observational cohort study; ROCT: retrospective observational cohort study; RCT: randomized controlled trial.

Study	Year	Type of study	Intubation	Extubation	Tracheostomy	Surgery
Stechmiller [11]	1994	POCT		15 (8%)		22 (12%)
Adam [12]	1997	POCT	34 (7.1%)	72 (15%)	32 (6.7%)	39 (8.1%)
McClave [13]	1999	POCT	NR			39%
Pinilla [15]	2001	RCT		11 (20%)		16 (29.1)
De Jonghe [14]	2001	POCT		29 (30.8%)		
Engel [16]	2003	POCT		26 (11%)		10 (4%)
Elpern [17]	2004	POCT				35.7%
Binnekade [18]	2005	ROCT				
O'Leary-Kelley [19]	2005	POCT	30% airway not specified			23.3%
Rice [20]	2005	POCT	15 (19%)			41 (51.9%)
Petros [21]	2006	POCT				
Reid [22]	2006	POCT	21% airway not specified			
O'Meara [23]	2008	POCT	25.8% airway not specified			5.2%
Kim [24]	2010	POCT	32 (25.8%) extubation/intubation		5 (4%)	15 (12.1%)
Kim [6]	2012	POCT				
Williams [26]	2013	B&A study	42 (2.7%)	395 (25.2%)	168 (10.7%)	181 (11.5%)
Yip [27]	2013	POCT				
Passier [25]	2013	ROCT	6 (5%)	6 (5%)	20 (16.5%)	68 (56.2%)
Peev [28]	2015	POCT	29		23	4
Segaran [30]	2016	B&A study				
Kozeniecki [29]	2016	ROCT		56 (28.3%)		2 (1%)
Chapple [5]	2016	POCT				
Wesselink [32]	2018	ROCT				
Lee [31]	2018	POCT	90+56			15 (4.5%)
Ritter [33]	2019	POCT		6 (15%)		6 (15%)
Salciute-Simene [3]	2021	POCT			21 (16%)	21 (16%)

Table 6 shows the patient-related factors causing ENIs. Frequently mentioned reasons are high gastric residual volumes and nasogastric tube dysfunction. Only three studies reported medication infusion as a reason for the ENI; however, it is unclear that medications were administered. Specifically, 11 (2.3%), four (1.5%), and 27 (21.8%) of the ENIs were reported in the studies by Adam et al. [12], Petros et al. [21], and Kim et al. [24], respectively, (data not shown in Table 5). Two studies showed that 57 (31%) and 44 (19%) of the ENIs were caused by gastrointestinal bleeding (not shown in Table 6) [11,16].

**Table 6:** Patient-related factors causing ENIs Values are displayed as percentage or as frequency and percentage (N (%)) ENI: enteral nutrition interruption; GRV: gastric residual volume; AL: anastomotic leakage; NGTB: nasogastric tube dysfunction; IAH: intra abdominal hypertension; ChT: chylothorax; B&A: before-and-after; POCT: prospective observational cohort study; ROCT: retrospective observational cohort study; RCT: randomized controlled trial

Study	Year	Type of study	Unstable hemodynamics	High GRV	Vomiting	Diarrhea	AL	NGTB	IAH	ChT	Ileus	Aspiration	Unknown
Stechmiller [11]	1994	POCT						49 (27%)			16 (9%)		8 (4%)
Adam [12]	1997	POCT		56 (11.7%)	50 (10.4%)	11 (2.3%)		45 (9.4%)	35 (7.3%)			26 (5.4%)	68 (14.2%)
McClave [13]	1999	POCT		45%				41%					
Pinilla [15]	2001	RCT			1 (1.8%)			4 (7.3%)	1 (1.8%)				18 (32.7%)
De Jonghe [14]	2001	POCT		26 (27.7%)				14 (14.9%)					
Engel [16]	2003	POCT		120 (51%)				17 (7%)					19 (8%)
Elpern [17]	2004	POCT	13.5%	11.5%				2.7%					11.2%
Binnekade [18]	2005	ROCT											
O'Leary-Kelley [19]	2005	POCT		21.7%				24.9%					
Rice [20]	2005	POCT	6 (7.6%)	5 (6.3%)				3 (3.8%)					10 (12.7%)
Petros [21]	2006	POCT		43 (18%) vomiting/high GRV		12 (4.8%)		11 (4.4%)					24 (9.9%)
Reid [22]	2006	POCT		14%				5%					
O'Meara [23]	2008	POCT	2.1%	9.7%				17.3%					
Kim [24]	2010	POCT	15 (12.1%)	8 (6.5%)	3 (2.4%)						2 (1.6%)	2 (1.6%)	15 (12.1%)
Kim [6]	2012	POCT											
Williams [26]	2013	B&A study		96 (6.1%)	70 (4.5%)	8 (0.5%)		129 (8.2%)				20 (1.3%)	199 (12.7%)
Yip [27]	2013	POCT		27 (38%)	2 (2.9%)	6 (8.4%)		5 (5.6%)					
Passier [25]	2013	ROCT											21 (17.4%)
Peev [28]	2015	POCT		10									
Segaran [30]	2016	B&A study											
Kozeniecki [29]	2016	ROCT	4 (2%)	15 (7.6%)	3 (1.5%)			20 (10.1%)	3 (1.5%)				83 (41.9%)
Chapple [5]	2016	POCT											
Wesselink [32]	2018	ROCT											
Lee [31]	2018	POCT	2 (0.6%)	12 (3.6%)	3 (0.9%)	2 (0.6%)	23 (6.9%)	3 (0.9%)	6 (1.8%)				82 (24.7%)
Ritter [33]	2019	POCT		2 (5%)	2 (5%)								24 (60%)
Salciute-Simene [3]	2021	POCT	26 (20%)	22 (17%)		13 (10%)	4 (3%)	4 (3%)	4 (3%)	1 (0.8%)	1 (0.8%)	1 (0.8%)	1 (0.8%)

Nutritional implications of enteral nutrition interruptions

Table 7 shows the number of nutritional assessment days and the effect of ENIs on feeding. Eleven studies reported on the nutritional implications of ENI, e.g., underfeeding, normofeeding, or overfeeding. As seen in Table 7, a significant number of patients are underfed, and overfeeding of patients was reported in only three studies [3,22,24].

**Table 7:** Amount of nutritional assessment days and the effect of ENIs on feeding Values are displayed as frequency (N) or as frequency and percentage (N (%)) ENI: enteral nutrition interruption; N: amount; B&A: before-and-after; POCT: prospective observational cohort study; ROCT: retrospective observational cohort study; RCT: randomized controlled trial

Study	Year	Type of study	Days assessed for adequacy of nutrition	Underfeeding (n)	Normofeeding (n)	Overfeeding (n)
Stechmiller [11]	1994	POCT	624	480 (76.9%)	144 (23.1%)	
Adam [12]	1997	POCT	1929	463 (24%)	1466 (76%)	
De Jonghe [14]	2001	POCT	484	245 (50.6%)	239 (49.4%)	
Engel [16]	2003	POCT	700	322 (46%)	378 (54%)	
Binnekade [18]	2005	ROCT	3526	1684 (47.8%)	1842 (52.2%)	
Petros [21]	2006	POCT	750		78.9%	
Reid [22]	2006	POCT	211	92 (43.6%)	70 (33.2%)	49 (23.2%)
Kim [6]	2012	POCT		62%	29%	9%
Segaran [30]	2016	B&A study	616	157 (25.5%)	459 (74.5%)	
Chapple [5]	2016	POCT	491	309 (62.9%)	182 (37.1%)	
Lee [31]	2018	POCT	1367			
Salciute-Simene [3]	2021	POCT	877	249 (28.4%)	337 (38.4%)	291 (33.2%)

Discussion

This systematic review aimed to give an update and overview of the evidence regarding EN adequacy in critically ill patients, in particular the frequency, causes, and nutritional implications of EN interruptions. Due to a lack of heterogeneous reporting of ENI’s and its causes, a meta-analysis was not performed.

In general, we can divide the ENI causes into three groups: patient-related factors, interventional procedures, and diagnostic procedures. Patient-related factors include, for example, an ileus, diarrhea, vomiting, aspiration, and high gastric residual volume. Interventional procedures are interventions involving the airways, namely intubation, extubation, tracheostomies, and other surgical procedures. Diagnostic procedures include radiology scans and endoscopies [3,26].

The most important problem is the inconsistent reporting of several pivotal factors in the nutritional management of critically ill patients in the ICU: 1) different methods are used for the evaluation of energy and/or protein requirements; 2) different criteria are used to determine an adequate intake; 3) in the case of an ENI, different definitions are used; 4) inadequate reporting of Prokinetic; and 5) the registration of how long an ENI exactly lasted. Besides the above-mentioned aspects, differences in hospital protocols can also contribute to these variations.

Regarding the evaluation of energy and protein requirements, there is no consensus among the included studies in this systematic review. Many methods are used: ASPEN guideline, Penn State equations, the Harris and Benedict formula, indirect calorimetry, and weight-biased formula of 25-30 kcal/kg daily [3,5,14,16,18,22,30,31].

Differences in energy goals, definitions of adequate intake (including underfeeding and overfeeding), and the number of ENI episodes were observed when comparing studies conducted in different countries. A cross-sectional prospective study conducted in Malaysia followed 77 patients during their ICU stay. Only 16 out of the 77 patients (20.7%) did not have any ENI, while the rest had one or more ENIs. In total, the study registered 72 ENI episodes [27]. A prospective observational study, conducted in the state of South Australia, followed 37 patients during their ICU stay. Patients had ENI on 309 of 491 days during their ICU stay [5]. Another study from Australia shows a decrease in ENI from 907 to 662. Between the measurement periods, at least 80% of the nursing and medical staff received training over a six-week period [26]. There is also a difference between the duration of ENI in the ICU. A recent study, conducted in Lithuania, shows a median duration of 12 hours (IQR 6-24 hours). The longest duration was observed in patient-related factors, namely a median of 22 hours (IQR 12-42 hours) [3]. Another study, conducted in Korea, shows the duration of ENI in patients who are adequately fed in group one and those who are underfed in group two. Patients are considered underfed if their total energy intake is <90%. The mean ENI duration for group one is 2.2 hours (±3.9), while for group two, it is 8.4 hours (±11.5) [6]. The above-mentioned examples might indicate that there are at least differences in hospital protocols, as well as in the definition and registration of ENI episodes.

Limitations

Despite this systematic review comprising 26 included studies, several limitations need to be addressed. In the current studies, inconsistent reporting of outcome measures was present, and therefore a meta-analysis was omitted. In the nutritional management of patients in the ICU, there is inconsistency in the reporting of several aspects, which makes the generalizability of results more difficult. Regarding adequate intake, ESPEN guidelines describe how to progress on the energy and protein target; however, since the wide inclusion of studies (also the inclusion of older studies), it is unclear how often the guideline principles are used [3]. Therefore, there might be bias in defining how much the patient is underfed, normofed, or overfed. Among these factors are the evaluation of energy and/or protein requirements, objectively determining an adequate intake, and finally reporting uniformity in the causes, frequency, and duration of ENIs.

## Conclusions

Although the nutritional management of critically ill patients in the ICU has improved drastically, ENIs are still a major problem in clinical practice. Due to variations in hospital protocols, ENI percentages and underfeeding as a result of ENI vary greatly. Future research needs to focus on uniformity in reporting the evaluation of energy and/or protein requirements, objectively determining an adequate intake, and reporting the causes, frequency, and duration of ENIs.
